# Native prey, not landscape change or novel prey, drive cougar (*Puma concolor*) distribution at a boreal forest range edge

**DOI:** 10.1002/ece3.11146

**Published:** 2024-04-01

**Authors:** Millicent V. Gaston, Andrew F. Barnas, Rebecca M. Smith, Sean Murray, Jason T. Fisher

**Affiliations:** ^1^ School of Environmental Studies University of Victoria Victoria British Columbia Canada

**Keywords:** anthropogenic development, boreal, camera traps, carnivore recovery, predator–prey interactions, range expansion

## Abstract

Many large carnivores, despite widespread habitat alteration, are rebounding in parts of their former ranges after decades of persecution and exploitation. Cougars (*Puma concolor*) are apex predator with their remaining northern core range constricted to mountain landscapes and areas of western North America; however, cougar populations have recently started rebounding in several locations across North America, including northward in boreal forest landscapes. A camera‐trap survey of multiple landscapes across Alberta, Canada, delineated a range edge; within this region, we deployed an array of 47 camera traps in a random stratified design across a landscape spanning a gradient of anthropogenic development relative to the predicted expansion front. We completed multiple hypotheses in an information‐theoretic framework to determine if cougar occurrence is best explained by natural land cover features, anthropogenic development features, or competitor and prey activity. We predicted that anthropogenic development features from resource extraction and invading white‐tailed deer (*Odocoileus virgianius*) explain cougar distribution at this boreal range edge. Counter to our predictions, the relative activity of native prey, predominantly snowshoe hare (*Lepus americanus*), was the best predictor of cougar occurrence at this range edge. Small‐bodied prey items are particularly important for female and sub‐adult cougars and may support breeding individuals in the northeast boreal forest. Also, counter to our predictions, there was not a strong relationship detected between cougar occurrence and gray wolf (*Canis lupus*) activity at this range edge. However, further investigation is recommended as the possibility of cougar expansion into areas of the multi‐prey boreal system, where wolves have recently been controlled, could have negative consequences for conservation goals in this region (e.g. the recovery of woodland caribou [*Rangifer tarandus caribou*]). Our study highlights the need to monitor contemporary distributions to inform conservation management objectives as large carnivores recover across North America.

## Graphical abstract

Cougar populations are rebounding in North America after decades of persecution. We used camera traps to examine factors influencing cougar expansion at a range edge in Alberta's northeast boreal forest. We found that native prey, specifically snowshoe hare, is the best predictor of cougar occurrence at this range edge. Potential cougar expansion has implications for this area of intense conservation concern due to industrial resource extraction and shifting community dynamics.

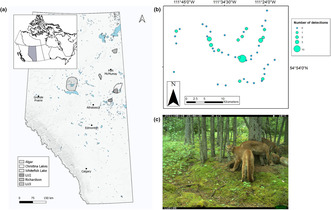

## 1 INTRODUCTION

In North America, several apex predator species are currently recovering and recolonizing parts of their former ranges after decades of persecution, overexploitation, and habitat alteration (Moss et al. 2016a; Cimatti et al., 2021; Engebretsen et al., 2021; Gantchoff et al., 2021; Pratzer et al., 2023). Habitat modification from anthropogenic landscape development – such as resource extraction and urbanization—alters mammal distributions, predator–prey interactions, and community compositions (Tattersall et al., 2020b; Wittische et al., 2021). Although population declines and range restrictions have been observed across taxa (Burgar et al., 2019; Fisher & Burton, 2018; Tattersall et al., 2020b; Nagy‐Reis et al., 2021; Wittische et al., 2021; Frey et al., 2022), shifting resource availability from anthropogenic development is facilitating geographic range expansions in some adaptable species that can exploit these novel landscapes (Fisher & Burton, 2021; Lanszki et al., 2022; Pattison et al., 2020; Tattersall et al., 2020a). Some large carnivores recovering in increasingly developed landscapes include grizzly bears (*Ursus arctos*; Clark et al., 2019; Clark et al., 2022; Mace et al., 2012; Pyare et al., 2004), black bears (*Ursus americanus*; Gantchoff et al., 2022), gray wolves (*Canis lupus*; Latham et al., 2011; Mech, 2017), and cougars (*Puma concolor*; LaRue & Nielsen, 2016; Moss, Alldredge, & Pauli, 2016b).

Cougars are solitary, opportunistic carnivores that inhabit a variety of habitat types and climates, from mountains to tropical rainforests to deserts (Comiskey et al., 2002; Guerisoli et al., 2021; Kertson et al., 2011; Monroy‐Vilchis et al., 2009; Robins et al., 2019). Prior to the late 1800s, cougars spanned from southeast Alaska in North America to southern Chile in South America (Jung & Merchant, 2005; Matte et al., 2013; Smereka et al., 2020). However, cougars have since been eradicated from much of their former range primarily due to declining ungulate prey and unregulated harvest (Anderson Jr. et al., 2009; Dickson & Beier, 2002; Gantchoff et al., 2021; Mallory et al., 2012; Morrison et al., 2015; Winkel et al., 2023). In recent decades, cougars have begun expanding east of their western range in North America (Gantchoff et al., 2021; LaRue et al., 2012; Mallory et al., 2012; Olson et al., 2021; Stoner et al., 2021) and have now been observed moving northward in the boreal forest (Anderson Jr. et al., 2009; Knopff, Webb, & Boyce, 2014b; Morrison et al., 2015; Winkel et al., 2023). Similar to other large carnivores, improved conservation management has played a major role in cougar recovery (Anderson Jr. et al., 2009; Gantchoff et al., 2022; Knopff, Webb, & Boyce, 2014b; LaRue & Nielsen, 2016); however, there are likely other ecological factors aiding re‐establishment.

Cougar distributions are partially structured by prey availability and accessibility (Knopff, Webb, & Boyce, 2014b; Maletzke et al., 2017; Monroy‐Vilchis et al., 2009). Cougar recovery has been partly facilitated by growing ungulate populations, including white‐tailed deer (*Odocoileus virgianius*), which have expanded their range extensively throughout North America in the last fifty years (Cooley et al., 2008; Darlington et al., 2022; Maletzke et al., 2017; Mallory et al., 2012; Winkel et al., 2023). The footprint of anthropogenic development from resource extraction, including features such as petroleum exploration seismic lines, provides early seral vegetation subsidies for ungulates (Fisher & Burton, 2018; Fuller et al., 2022; McKay & Finnegan, 2022; Rea, 2003). Increased forage from regenerating natural landscape features, combined with milder winters, have facilitated white‐tailed deer invasions in formerly deer‐limited regions (Angert et al., 2020; Dawe & Boutin, 2016; Pattison et al., 2020; Tattersall et al., 2020b). Thus, cougars can benefit from industrialized landscapes, without significant human presence, due to increased accessibility of ungulate and small‐bodied prey (Knopff, Knopff, et al., 2014a; Moss, Alldredge, & Pauli, 2016b; Smith et al., 2016; Smereka et al., 2021; Stoner et al., 2021). These trends are observed in other large carnivores in anthropogenically modified landscapes (Boydston et al., 2003; Gantchoff et al., 2022; Hebblewhite & Merrill, 2008; Martin et al., 2010). Cougars also display a degree of habitat flexibility in developed landscapes, which allows them to use linear features such as seismic lines as travel corridors, along with edge habitat that provides optimal cover for ambush hunting strategies (Dickson et al., 2005; Kertson et al., 2011; Knopff, Knopff, et al., 2014a; Maletzke et al., 2017; Morrison et al., 2014, 2015; Moss, Alldredge, Logan, & Pauli, 2016a; Pattison et al., 2020; Robins et al., 2019).

In the last century and more, cougars have been largely absent from the northeast boreal forest of Alberta, Canada—a highly industrialized landscape dominated by forestry and energy sectors, creating networks of linear and polygonal features for logging, petroleum exploration, and extraction, alongside transportation and urban infrastructure (Fisher & Burton, 2018). While previously constrained to their northern core range in mountain landscapes and areas of western North America, telemetry data and increased harvest rates have revealed cougar dispersal and re‐colonization in some areas of eastern Alberta, including Cypress Hills Interprovincial Park (Anderson Jr. et al., 2009; Knopff, Knopff, et al., 2014a; Knopff, Webb, & Boyce, 2014b; Morrison et al., 2014; Smereka et al., 2021). The northern boreal forest plains east of the Rocky Mountains are thought to be outside their historic range (Winkel et al., 2023). However, there have been increasing reports of cougar sightings, roadkill incidents, and livestock depredation events in Alberta's northern communities including Athabasca, Grand Prairie, and Fort McMurray (Anderson Jr. et al., 2009; Knopff, Webb, & Boyce, 2014b), as well as reports in the Yukon and Northwest Territories (Gau et al., 2001; Jung & Merchant, 2005). If cougars follow the influx of deer into a novel, industrialized landscapes in the boreal forest plains, increased cougar occurrence at this northeast range edge could have implications as predator–prey dynamics shift (Knopff, Webb, & Boyce, 2014b; Mallory et al., 2012; Winkel et al., 2023).

We propose that landscape alteration from rapid industrial development (i.e. the footprint of human development, not the physical presence of humans) and subsequent white‐tailed deer invasions are driving a potential cougar population expansion at a northeastern range edge. We sought to determine the best‐supported factors driving cougar occurrence in the boreal using camera traps (Burton et al., 2015). Camera traps are a useful tool for studying elusive, far‐ranging mammals that live in low densities and have been used effectively for cougars (Alexander & Gese, 2018; Guarda et al., 2017; Loonam et al., 2021; Procko et al., 2022, 2023). We collected data from a camera array deployed at the edge of the cougar's northeastern range in Alberta, Canada, relative to the proposed expansion front. We weighed evidence for the competing hypotheses that cougar occurrence is best explained by: (1) natural landcover; (2) anthropogenic development features; and (3) the presence of other mammal species, including competitor and prey activity; using model selection in an information‐theoretic framework (Burnham & Anderson, 2002). We predict that cougar occurrence is driven by the relative proportion of anthropogenic development features and the availability of large, non‐native prey: the relative activity of white‐tailed deer.

## 2 STUDY AREA

Alberta's boreal forest is a mosaic dominated by trembling aspen (*Populus tremuloides*), white spruce (*Picea glauca*), and black spruce (*P. mariana*) interspersed with lakes, rivers, fens, and bogs (Roberts et al., 2022). Within this sits Alberta's oil sands region (OSR), which has extensive development features from active oil and gas extraction (seismic lines, well‐sites, pipelines), forestry harvest (cut blocks), transportation (roads, railways), and other industrial and urban infrastructure (Burgar et al., 2019; Frey et al., 2022). The boreal landscape supports a diverse range of mammalian species, including apex predators such as gray wolf and black bear, mesocarnivores such as coyote (*Canis latrans*), lynx (*Lynx canadensis*), and various mustelids (*Mustela* spp.), and prey species such as white‐tailed deer, moose (*Alces alces*), woodland caribou (*Rangifer tarandus caribou*), and snowshoe hare (*Lepus americanus*).

**FIGURE 1:**
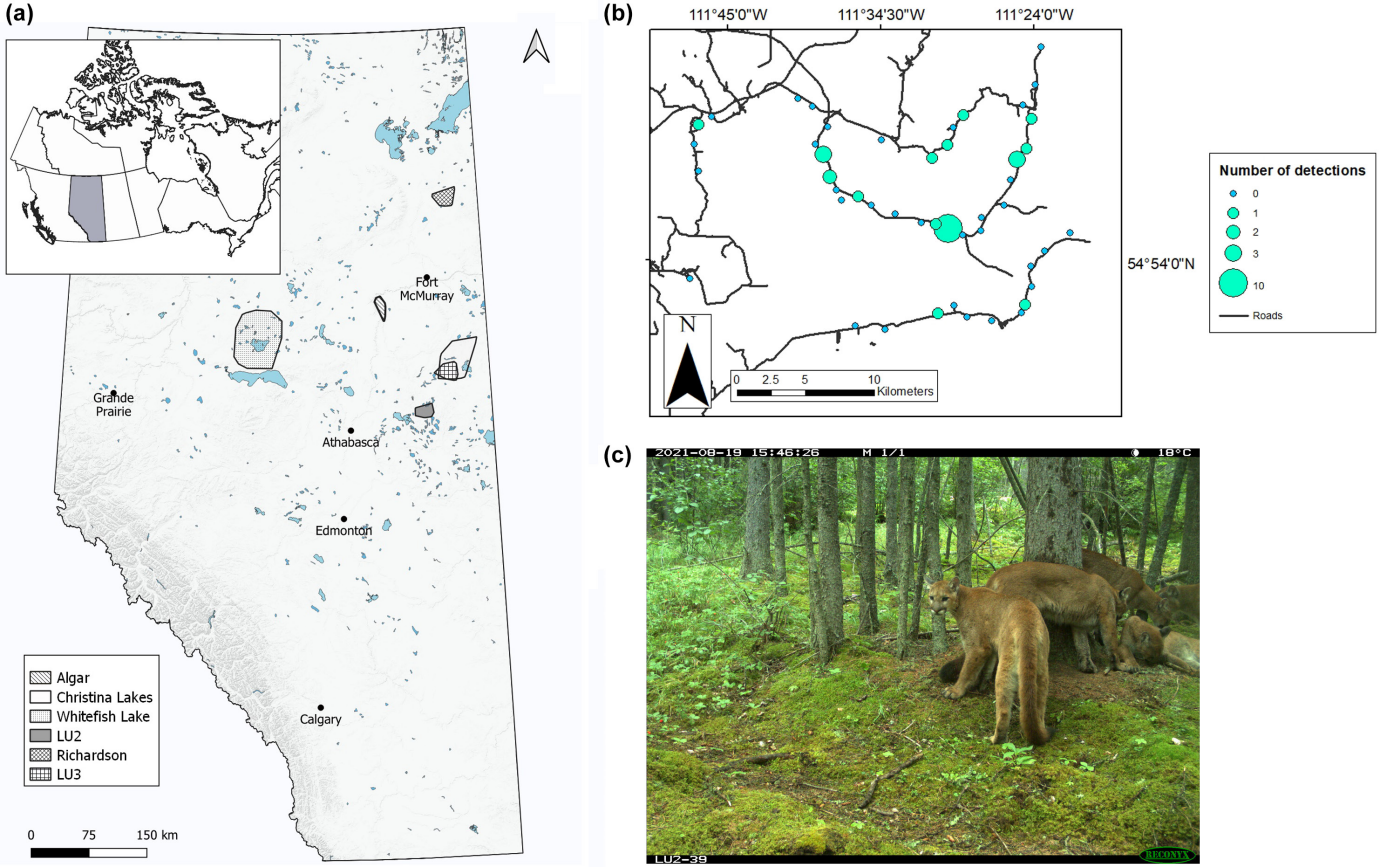
(a) Map of six camera trap arrays deployed across the oil sands region (OSR) in Alberta, Canada. (b) Distribution of camera traps across OSM Landscape Unit 2 (LU2), the study area. Each circle represents a camera location (*n* = 47) and increasing circle size corresponds to a higher number of cougar detections. (c) Example image of multiple cougars (*n* = 5) on a single camera.

From 2011 to the present our research program has been monitoring mammal communities across the region using camera traps, partly under the Joint Canada‐Alberta Oil Sands Monitoring Program (OSM). The OSM is a multi‐partner endeavor that includes the provincial and federal Governments of Alberta and Canada, along with Indigenous community‐based monitoring programs (Roberts et al., 2022). The OSM design aims to capture multiple mammal species, including everything from snowshoe hares to moose in size, across a gradient of natural and anthropogenic heterogeneity. At six camera arrays spread over 15,000 km^2^ throughout Alberta's OSR, we examined cougar occurrences (Figure 1a). Five of these arrays captured very little cougar presence (<0.05 naïve occupancy in each array). The sixth array, OSM Landscape Unit 2 (LU2; 500 km^2^, *n* = 47 cameras deployed from 2021 to 2022), contained multiple cougar detections (Figure 1b), so we examined cougars within this array.

## 3 METHODS

### 3.1 Sampling design

OSM Landscape Unit 2 (LU2), is a polygon over 500 km^2^ of boreal forest, 25 km northeast of Lac La Biche, Alberta, Canada (Figure 1a), located partially on Heart Lake First Nation territory. To examine factors contributing to cougar occurrence at this range edge, this landscape polygon was divided into 2 km^2^ hexagonal grid cells in ArcGIS v 10.3 (Environmental Systems Research Institute, Redlands, CA) and stratified into upland (cells comprised of >50% deciduous forest) and lowland (cells comprised of >50% coniferous forest) areas, as these are the dominant landcover classes in the study area. The 2 km^2^ cell size employed for our camera sampling design was designed as a compromise across a wide range of wildlife body sizes and home range sizes (Holling, 1992). The resulting hexagonal grid cells were further constrained to be within 100 m of roads for available ground access. The constraint means that sites were closer to roads at small scales than sites selected at random, as access makes roads and trails more highly represented at small (<250 m) spatial scales, but this effect disappears at larger scales (>1 km) as development is widespread across the landscape (sensu Fisher & Burton, 2018; Fisher et al., 2020; Fisher & Ladle, 2022). From these constrained grid cells, an equal number of cells from each of the two strata were randomly selected as candidate sites. This resulted in a total of 60 candidate sites, an over‐selection to account for inaccessibility not identified prior to field deployment. Of the 60 candidate sites, we were able to access and deploy cameras at 47 sites (Figure 1b).

One infrared remote camera (Reconyx™ PC900 model; Reconyx, Holmen, WI) was deployed in each accessible grid cell, at least 100 m from any roads, at a sampling site (*n* = 47), which is defined as the area included in the camera detection zone (Burton et al., 2015). At each sampling site, a camera was placed approximately 1.5 m up a tree along an active wildlife trail, facing another tree baited with commercial scent lure (O'Gorman's™ Long Distance Call) and its surrounding area, to maximize detection probability, i.e., to maximize the probability of an animal being in the camera's frame of view, sensu Fisher and Bradbury (2014). Cameras were programmed to take 1 photo when movement was detected by the infrared sensor, using a high sensor sensitivity with no delay between detections, and a ‘timelapse’ photo captured at the same time daily to verify camera operability. Each camera was placed at an approximate minimum distance of 1 km away from any other camera to facilitate independence among sample sites, albeit recognizing that predators with large home ranges may occur on adjacent cameras. We sampled 47 sites in total over a sampling period of approximately one year. We deployed 47 cameras in July 2021, and retrieved 22 in February 2022 (8 months) and the remaining 25 in September 2022 (14 months).

### 3.2 Image review and relative activity indices

**FIGURE 2:**
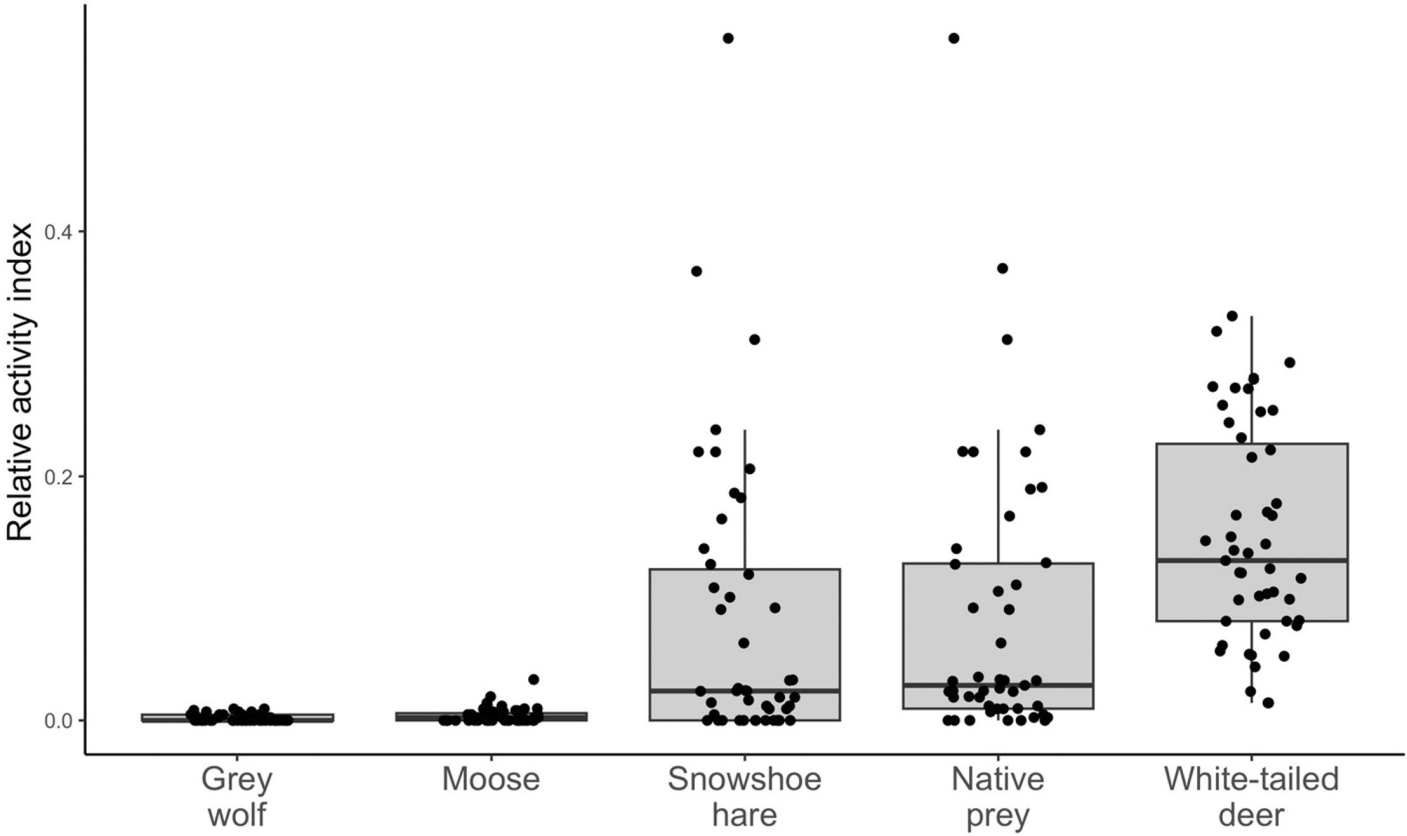
Relative activity indices at each camera site (*n* = 47) for potential prey and competitor predator species, including gray wolf (*Canis lupus*), moose (*Alces alces*), snowshoe hare (*Lepus americanus*), native prey (moose and snowshoe hare combined), and white‐tailed deer (*Odocoileus virgianius*). Activity indices were calculated by summing all independent detections (defined by events occurring a minimum of 30 min apart) for each species and dividing by the total number of active camera days, to create a metric of species activity on the landscape.

Trained personnel used Timelapse Image Analyzer 2.0 (Greenberg et al., 2019) to manually review and identify animals in each image from remote camera traps. As multiple subsequent images of the same species on the same camera likely constitute the same individual, we defined repeat animal detections as independent events if they occurred a minimum of 30 minutes apart. We quantified independent events for cougars, non‐native prey species (white‐tailed deer), native prey species (moose and snowshoe hare), and the primary competitor/predator (gray wolf). Because we were explicitly interested in the relationship between cougar occurrence relative to native vs. non‐native prey activity, we grouped moose and snowshoe hare into one native prey variable. To account for differences in camera deployment duration we used a relative activity index as a measure of predator and prey occurrence (to be used as explanatory variables in models), calculated by summing the total number of independent detections for each species and dividing by the total number of active camera days, resulting in an index of activity for each species (Figure 2). Thus, activity indices were used as a measure of time allocation to determine patterns of observed use frequency (i.e. where animals tend to spend time) for each species. We aimed to avoid detection failure (false absences) by using scent lures and game trails to improve the detectability of highly mobile animals and accounted for camera failure by programming cameras to take a single photo each day to ensure operability across the entire sampling period. Thus, we assume non‐detections to be true absences, i.e., signals of site usage frequency, and therefore did not employ an occupancy modeling framework. While occupancy modeling assumes absences to be errors due to imperfect detection (MacKenzie et al., 2003), we treated non‐detections as ecological signals of absence (temporary emigration) from a site, wherein more absences are suggestive of lower site value to the species of interest, sensu Fisher & Burton, 2018. Vagile animals spend more or less time at a site for a variety of ecological reasons, such as risk and reward trade‐offs and optimal foraging in patchy environments, among other environmental reasons (Fretwell, 1969, 1972; Pyke, 1984), which makes absences from a site an important part of the ecological signal. Thus, non‐detections reflect the biological process we are trying to capture, rather than error resulting from the observational process (Fisher & Burton, 2018).

### 3.3 Quantifying natural and anthropogenic landscape features

**FIGURE A1:**
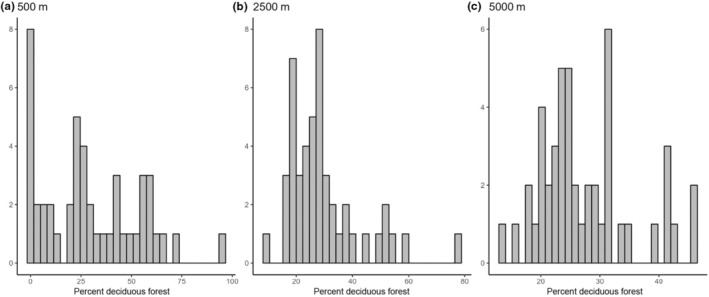
Histograms of the percentage of deciduous forests at camera sites (*n* = 47) collected at (a) 500 m, (b) 2500 m, and (c) 5000 m.

**FIGURE A2:**
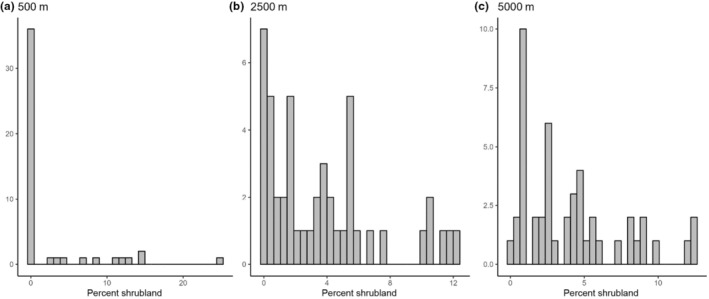
Histograms of the percentage of shrublands at camera sites (*n* = 47) collected at (a) 500 m, (b) 2500 m, and (c) 5000 m.

**FIGURE A3:**
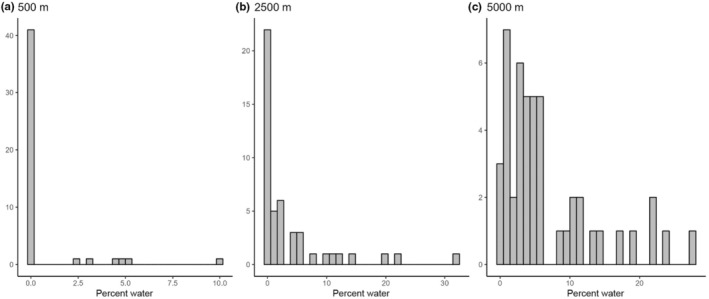
Histograms of the percentage of water at camera sites (*n* = 47) collected at (a) 500 m, (b) 2500 m, and (c) 5000 m.

Species habitat selection can vary depending on the spatial scale in which we quantify available resources (Jackson & Fahrig, 2012). To investigate cougar presence, we constructed models (see below) at three different spatial scales: small (500 m), intermediate (2500 m), and large (5000 m). We did not find any other well‐supported scale justifications specific to cougars in the literature. Thus, we created a 500, 2500, and 5000 m radius buffer around each camera site to quantify natural and anthropogenic landscape data at each scale. We were not concerned with overlapping buffers as spatial scale increased. It has been shown that landscape overlap does not increase spatial autocorrelation or violate assumptions of independence between sites, because overlapping landscapes are distinct from pseudoreplicaton, especially if predictor variables are scaled, as in this case (Zuckerberg et al., 2012, 2020). Using a LandSat digital map inventory from the Alberta Biodiversity Monitoring Institute (Alberta Biodiversity Monitoring Institute, 2010a), we quantified the proportion of three natural landcover classes within each site buffer: deciduous forest, shrubland, and water (Figures A1, A2, and A3). We chose these natural landcover variables due to their association with prey species of interest (i.e. valuable forage habitat for ungulates; Cairns & Telfer, 1980; Faison et al., 2016), and due to cougar association with riparian areas (Dickson et al., 2005; Dickson & Beier, 2002; Kertson et al., 2011; Pattison et al., 2020; Smereka et al., 2020). The study area is a relatively flat landscape (i.e. not rugged), which is why we did not include the terrain ruggedness index (TRI) as an explanatory variable in our analyses, although we are aware of its importance for cougars (Dickson et al., 2005).

**FIGURE A4:**
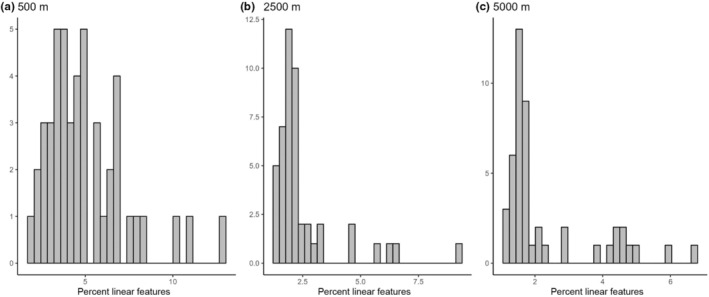
Histograms of the percentage of linear features (summed proportion of roads, seismic lines, railways, pipelines, trails, and transmission lines) at camera sites (*n* = 47) collected at (a) 500 m, (b) 2500 m, and (c) 5000 m.

**FIGURE A5:**
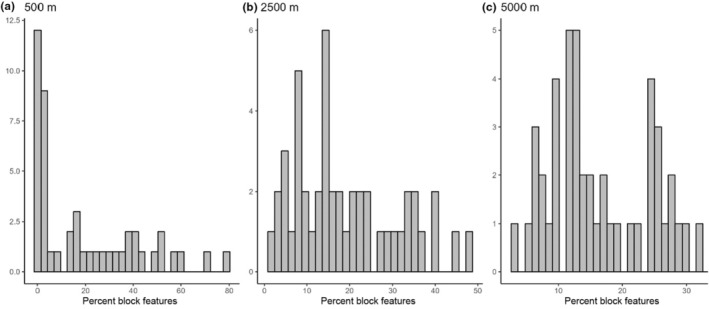
Histograms of the percentage of block features (summed proportion of well‐sites, borrow pits, harvest areas, and residential areas) at camera sites (*n* = 47) collected at (a) 500 m, (b) 2500 m, and (c) 5000 m.

Using the Alberta Biodiversity Monitoring Institute's Human Footprint Inventory (Alberta Biodiversity Monitoring Institute, 2010b), we measured the total proportions of both linear and polygonal (herein block) anthropogenic features within each site buffer. We created a single linear features variable (Figure A4) by summing the total proportion/area of roads, seismic lines, railways, pipelines, trails, and transmission lines within each buffer, separately for all three spatial scales. Similarly, we created a single block features variable (Figure A5) by summing the total proportion/area of well‐sites, borrow pits, harvest areas, and residential areas within each buffer for each spatial scale.

### 3.4 Statistical analysis

**FIGURE A6:**
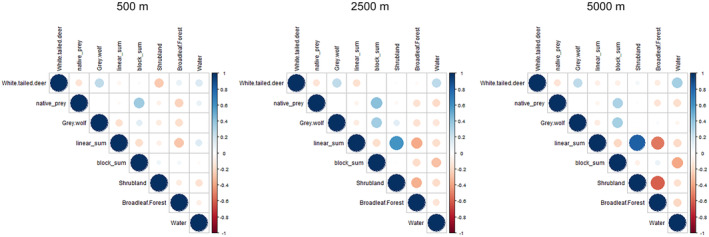
Pearsons correlation plots for unscaled continuous variables were used to build a candidate set of generalized linear models at each spatial scale (500, 2500, and 5000 m). Found a significant correlation (Pearson's *r* > .7) between linear features and shrubland at the 5000 m spatial scale (*r* = .82, *p* < .0001).

We constructed nonmutually exclusive competing hypotheses based on the primary drivers of cougar occurrence and represented them as a candidate set of generalized linear models examining cougar presence at camera sites (1 = present, 0 = absent), which we ran separately at a 500, 2500, and 5000 m spatial scale. Prior to model construction, we tested for correlations (Pearson's correlation coefficient, *r* > .7; Zuur et al., 2010) between all covariates at each scale and found a significant correlation between linear features and shrubland at the 5000 m scale (*r* = .82, *p* < .0001) (Figure A6). We, therefore, excluded the shrubland variable from any models at the 5000 m scale analysis.

**FIGURE A7:**
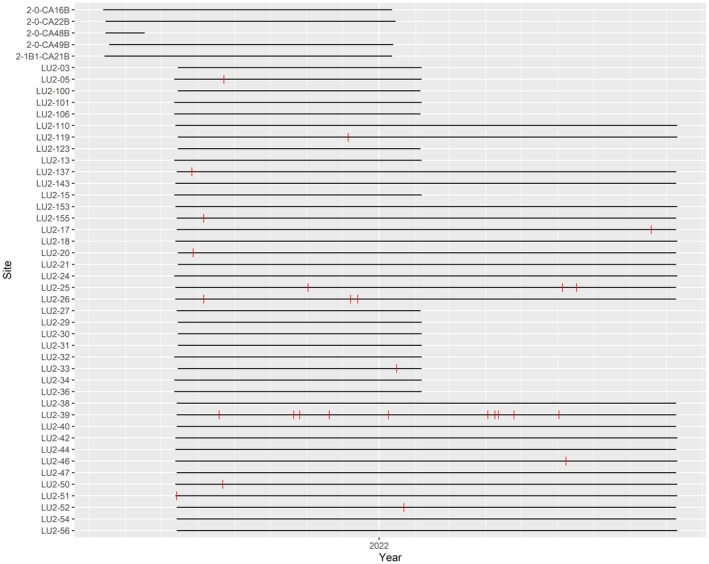
Camera operability across 2022 (designated by the horizontal black lines) for each camera (*n* = 47) in the OSM Landscape Unit 2 (LU2) study area in northeastern Alberta, Canada. Red dashes represent cougar detections at a camera.

We scaled all continuous variables to facilitate comparisons between β coefficients (effect sizes) and improve maximum likelihood estimation. We limited each model to a maximum of two covariates due to the limited sample size of sites with cougar presence (Austin & Steyerberg, 2017). We weighed the evidence for each model in an information‐theoretic framework based on Akaike's Information Criterion corrected for small sample sizes (AIC_c_) and determined effect sizes of model parameter β estimates (±SE). We calculated AIC_c_ weights for each of the models to determine which competing model was best supported among the candidate set at each scale (Burnham & Anderson, 2002). All models were fit with maximum likelihood, assuming a binomial distribution and using a clog log link function. We modeled cougar occurrence against: (1) natural landcover variables, including deciduous forest, shrubland, and water; (2) anthropogenic development features, including linear and block features; and (3) co‐occurrence of prey (native and non‐native) and competitor species, including white‐tailed deer, moose, snowshoe hare, and wolf; and we included a null model at each scale (Table 1). We did not include the water model at the 500 m scale analysis as this model did not converge at this scale due to a lack of representation of this feature. We did not include camera sampling effort as a fixed effect in models due to data limitations, but we were not overly concerned as cameras were broadly similar in operational days across sampling sites (Figure A7).

**TABLE 1:** List of the candidate set of competing models used to predicted cougar (*Puma concolor*) occurrence in northeastern Alberta, Canada.

Model	# of covariates	Covariates
Null	—	—
Natural Landcover	2	Proportion deciduous forest + Proportion shrubland^a^
Water^b^	1	Proportion water
Linear	1	Summed proportion roads + seismic lines + transmission lines + pipelines + railways
Block	1	Summed proportion well sites + harvest areas + residential areas + borrow pits
WTD	1	White‐tailed deer activity index
Native Prey	1	Summed moose + Snowshoe hare activity indices
Total Prey	2	Native prey + White‐tailed deer activity index
Wolf	1	Wolf activity index

^a^ Proportion shrubland covariate was not included in the Natural Landcover model at the 5000 m scale.

^b^ Water model was not included at the 500 m scale.

Model validation was performed for the best‐performing model at each spatial scale using a 10‐fold stratified cross‐validation. We used each top model to generate predicted marginal means for each fixed effect within that model. All statistical analyses were performed, and figures were generated, in the program R, using packages *dplyr* for data management (Wickham & Francois, 2015), *ggplot2* for data visualization (Wickham, 2016), *glmmTMB* for model construction (Magnusson et al., 2017), *ggeffects* for model predictions (Lüdecke, 2018), and *caret* for model validation (Kuhn, 2012; v. 4.1.2; R Core Team, 2018).

## 4 RESULTS

**FIGURE A8:**
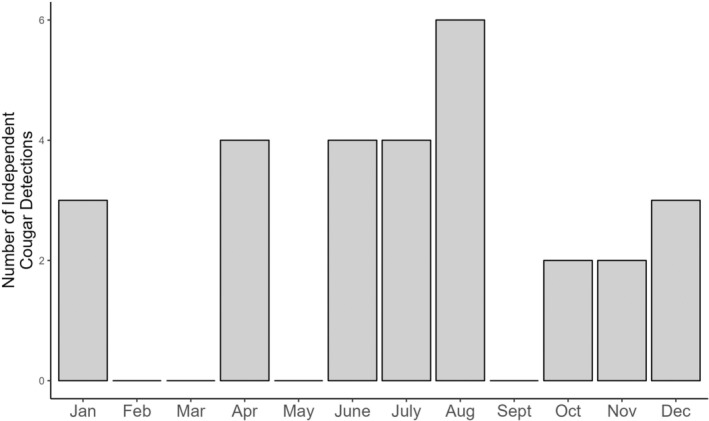
Independent detections of cougars by month across all cameras (*n* = 47) in the OSM Landscape Unit 2 (LU2) study area in northeastern Alberta, Canada.

Of the 47 cameras deployed in our study area, each camera was operating on the landscape for an average of 310.17 ± 121 active camera days (Figure A7). Cougars were detected at 14 out of 47 camera sites (30% of sites), with a total of 28 independent cougar detections over the 8‐to‐14‐month sampling period (Figure 1b). Examining the spread of independent cougar detections shows cougars were detected throughout the sampling period, across all seasons (Figure A8). Of the 14 sites where cougars were detected, there were multiple detections at 4 camera sites (Figure 1b), with a maximum of 5 individual cougars captured in some images (Figure 1c). For predator and prey explanatory variables, there were 2330 independent white‐tailed deer detections (detected at 100% of sites), 1251 independent snowshoe hare detections (74% of sites), 57 independent moose detections (51% of sites), and 35 independent gray wolf detections (38% of sites).

**FIGURE 3:**
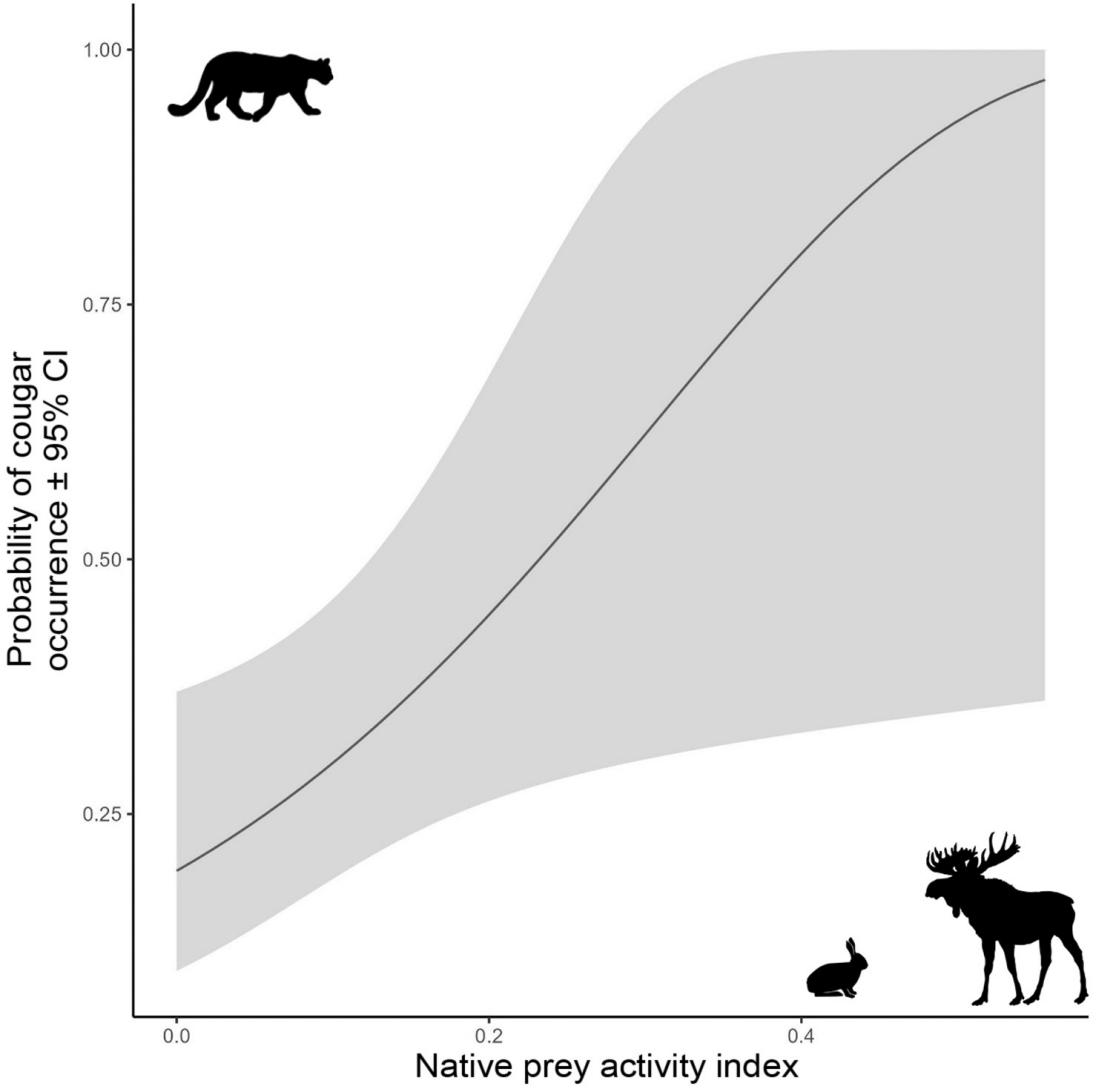
Predicted relationship between the probability of cougar (*Puma concolor*) occurrence (±95% confidence intervals) and the best‐supported model at all spatial scales, the relative activity of native prey (summed relative activity of moose, *Alces alces*, and snowshoe hare, *Lepus americanus*).

The native prey model, composed of the summed relative activity of moose and snowshoe hare, best‐predicted cougar occurrence on this landscape (500 m: AIC_cw_ = 0.39; 2500 m: AIC_cw_ = 0.44; 5000 m: AIC_cw_ = 0.42, Table 2). Native prey had a significant, positive association with cougar occurrence, and since independent detections of animals at camera sites do not vary with spatial scale (i.e. native prey is a scale‐invariant predictor), the size and direction of effect were identical across all three scales (*β* = 0.5815 ± 0.2679, *p* < .001, Table A1). Notably, snowshoe hares had a much higher activity index than moose (Figure 2). As estimates were identical for all three spatial scales, for simplicity we provide a single predictive plot of cougar occurrence related to native prey activity (Figure 3). The native prey model estimates an increasing probability of cougar occurrence at camera sites with increasing native prey activity, but notably, we found large confidence intervals with native prey values >0.2 due to a paucity of data past this range. Thus we caution interpretation of these findings at higher native prey values. Cross‐validation of the native prey model showed a high predictive accuracy (75.2%), but a low kappa coefficient (0.18), indicating only slight agreement between model predictions and out‐of‐sample data when accounting for chance alone (Landis & Koch, 1977). However, this is not unexpected given the small sample size of cougar occurrences at cameras (*n* = 14). We estimated Cook's Distance for potential outliers in the native prey model and found no points of influence.

**TABLE 2:** Model selection results for the candidate set of generalized linear models examining cougar (*Puma concolor*) occurrence in the northeast boreal forest of Alberta, Canada, at a 500, 2500, and 5000 m scale.

Scale	Model	AIC_c_	∆AIC_c_	AIC_cw_	−2LL	df
500 m	**Native Prey**	**56.55**	**0.00**	**0.39**	**−26.14**	**2**
	**Total Prey**	**57.93**	**1.38**	**0.19**	**−25.69**	**3**
	**Block**	**58.03**	**1.48**	**0.18**	**−26.88**	**2**
	Null	59.34	2.79	0.10	−28.63	1
	Linear	60.96	4.41	0.04	−28.34	2
	WTD	61.27	4.72	0.04	−28.50	2
	Wolf	61.52	4.97	0.03	−28.63	2
	Natural Landcover	61.61	5.06	0.03	−27.53	3
2500 m	**Native Prey**	**56.55**	**0.00**	**0.44**	**−26.14**	**2**
	**Total Prey**	**57.93**	**1.38**	**0.22**	**−25.69**	**3**
	Null	59.34	2.79	0.11	−28.63	1
	Linear	60.43	3.88	0.06	−28.08	2
	WTD	61.27	4.72	0.04	−28.50	2
	Block	61.40	4.85	0.04	−28.56	2
	Water	61.52	4.97	0.04	−28.62	2
	Wolf	61.52	4.97	0.04	−28.63	2
	Natural Landcover	62.66	6.10	0.02	−28.05	3
5000 m	**Native Prey**	**56.55**	**0.00**	**0.42**	**−26.14**	**2**
	**Total Prey**	**57.93**	**1.38**	**0.21**	**−25.69**	**3**
	Null	59.34	2.79	0.10	−28.63	1
	Natural Landcover	60.68	4.13	0.05	−28.20	2
	Linear	60.80	4.24	0.05	−28.26	2
	Block	61.07	4.52	0.04	−28.40	2
	WTD	61.27	4.72	0.04	−28.50	2
	Water	61.47	4.91	0.04	−28.60	2
	Wolf	61.52	4.97	0.04	−28.63	2

*Note*: Bolded models indicate those within ∆ 2 AIC_c_ of a top model.

At each spatial scale, the total prey model was competitive with the top model based on AIC_c_, (500 m: ∆AIC_c_ = 1.38, AIC_cw_ = 0.19; 2500 m: ∆AIC_*c*_ = 1.38, AIC_cw_ = 0.22; 5000 m: *∆*AIC_*c*_ = 1.38, AIC_cw_ = 0.21, Table 2). However, the total prey model likely contains an uninformative parameter (WTD activity index; *β* = 0.2725 ± 0.2845, *p* = .338) and thus was not considered further (Arnold, 2010; Burnham & Anderson, 2002). At the 500 m scale, the block features model was competitive with the top model, and we found a positive relationship between increasing block features and cougar occurrence (*∆*AIC_*c*_ = 1.48, AIC_cw_ = 0.18, Table 2; *β* = 0.4705 ± 0.2423, *p* = .052); however, the block model was not considerably different from the null model at the 500 m scale (Table 2). None of the other candidate models were better at explaining cougar occurrence than the null model.

## 5 DISCUSSION

Native prey activity best explains cougar occurrence in this area of Alberta's northeast boreal forest. The likelihood of a cougar occurring on the landscape was positively associated with the availability of native prey, driven predominantly by the relative activity of snowshoe hare. However, it is unclear whether this is a direct effect of snowshoe hare presence or a latent association between cougars and hare. Counter to our predictions, cougars did not have a strong association with non‐native white‐tailed deer activity or the relative proportion of anthropogenic development features on the landscape.

Like many apex predators, cougars are partially structured by prey populations (Monroy‐Vilchis et al., 2009; Blecha et al., 2018; Schmidt et al., 2018; Bhandari et al., 2021; Smereka et al., 2021). We infer cougar occurrence at this observed northeast boreal range edge is strongly associated with the relative activity of snowshoe hare. Although ungulates are the primary prey source for cougars across North America, cougars are opportunistic hunters that exploit alternative prey when available (Clark et al., 2014; Guerisoli et al., 2021; Moss, Alldredge, Logan, & Pauli, 2016a; Moss, Alldredge, & Pauli, 2016b; Smith et al., 2016). Female cougars raising offspring are especially reliant on small‐bodied prey (Stoner et al., 2021), such as hares, and will select habitats that provide opportunistic hunting opportunities to support high‐energetic demands (Clark et al., 2014; Smereka et al., 2021). Subadult cougars, which were captured on our cameras (Figure 1c), are also reliant on small‐bodied prey, likely due to hunting inexperience (Knopff et al., 2010; Moss, Alldredge, & Pauli, 2016b). Further, cougars are more likely to select for small‐bodied prey items in developed landscapes, especially females with kittens, due to increased prey availability and decreased handling time (due to smaller body sizes), allowing for increased vigilance (Moss, Alldredge, & Pauli, 2016b; Smith et al., 2016; Stoner et al., 2021). In the boreal forest, snowshoe hares are experiencing bolstering effects from human landscape development, as they exploit increased understorey cover in young‐ and mid‐successional forest stands following harvest (Fisher & Wilkinson, 2005; St‐Laurent et al., 2008). Stoner et al. (2021) found that, in developed landscapes, cougars select resource extraction features that provide edge habitat for ambush hunting, ungulate‐attracting forage, and increased presence of alternative small‐bodied prey. We did not detect a strong relationship between cougars and anthropogenic development, besides a weak positive association with block features (i.e. polygonal features from development including well‐sites, borrow pits, harvest areas, and residential areas) at our smallest spatial scale. However, cougars may be experiencing an indirect benefit from human development features due to increased prey accessibility, especially that of small‐bodied alternative prey sources, as seen in other large carnivores (Boydston et al., 2003; Hebblewhite & Merrill, 2008; Knopff, Knopff, et al., 2014a; Moss, Alldredge, & Pauli, 2016b). Future work should examine the pathways between anthropogenic development and small‐bodied prey, and the ultimate downstream effects on cougar occurrence.

The images captured of multiple subadult cougars in our study across all seasons indicate that there may be resident individuals breeding in this area (Figure 1c; Figure A8). Female cougars have smaller home range sizes and do not disperse long distances compared to males, which raises the possibility that the cougars captured on our cameras may be established near or within the focal study area, rather than just transient occurrences (Anderson Jr. et al., 2009; Knopff et al., 2010; Knopff, Webb, & Boyce, 2014b; Mallory et al., 2012; Smereka et al., 2021). Although this is speculative based on camera images, few studies to our knowledge have considered cougar presence at this boreal range edge, and with increasing population densities and extensive home range sizes, increased cougar presence at this range edge may be expected as cougars recover from past persecution (Anderson Jr. et al., 2009; Smereka et al., 2021). As cougar populations rebound across eastern Alberta, cougars have the potential to keep moving north into previously (or at least recently) unoccupied boreal forest landscapes where there are suitable forage opportunities to support breeding populations (Winkel et al., 2023). However, further monitoring is needed to determine whether these factors could contribute to cougar expansion in the northeast boreal forest as the landscape and community continue to change.

The association between cougars and native prey in this region has implications for current predator–prey dynamics in the boreal forest. Woodland caribou (*Rangifer tarandus caribou*) are experiencing steep declines due to habitat loss from industrial activity, which is amplified by increased wolf predation (Ehlers et al., 2014, 2016; Latham et al., 2011; Laurent et al., 2021; Nagy‐Reis et al., 2021; Spangenberg et al., 2019; Tattersall et al., 2020a). However, government‐mandated wolf control, employed since 2005, has removed hundreds of wolves from these landscapes (Alberta Environment and Parks, 2017). Although we did not find a strong relationship between wolf activity and cougar occurrence in the study landscape, we believe this warrants further investigation. While caribou were not detected in our focal study area (LU2), there are caribou present further northeast in a neighboring array (LU3). Thus, if cougars expand north, they may have the ability to fill the dominant apex predator role left by systematic wolf control via the open niche hypothesis. This could have subsequent consequences for attempted rebolstering of caribou populations (Anderson Jr. et al., 2009), although we recognize that our results do not directly support this. A similar phenomenon was observed in Nevada's Great Basin Desert, where historic black bear extirpation, coupled with increasing mule deer (*Odocoileus hemionus*), allowed for expansive cougar population growth in the 20th century (Engebretsen et al., 2021). Furthermore, the apparent‐competition (alternative prey) hypothesis predicts increased predation of caribou in multi‐prey systems (Apps et al., 2013; Holt et al., 1994; Robinson et al., 2002). Cougars in British Columbia, supported by deer and elk (*Cervus canadensis*) populations, are one of the main sources of caribou mortality, as alternate prey sources increase the likelihood of cougar‐caribou encounters (Apps et al., 2013; Kinley & Apps, 2001; Leech et al., 2017). Thus, possible cougar northern range expansion could have negative implications for Alberta's woodland caribou populations, with the potential to shift other community dynamics (Anderson Jr. et al., 2009; Boutin et al., 2012; Knopff, Webb, & Boyce, 2014b; McKay & Finnegan, 2022). Although our results do not directly support this, we believe it is worth consideration as the northern boreal community undergoes rapid change in the face of shifting species distributions, anthropogenic development, and climate change. In addition, changing cougar distributions have implications for humans, with the chance of human‐cougar conflict increasing in northern rural communities (Anderson Jr. et al., 2009; Morrison et al., 2014; Blecha et al., 2018; Robins et al., 2019). Further monitoring in the northeastern regions of Alberta is required for cougar management and conservation objectives as this apex predator potentially expands across the province.

Counter to our predictions, the relative activity of white‐tailed deer did not carry strong explanatory power in predicting cougar occurrence across spatial scales compared to native prey. Non‐native white‐tailed deer populations are sustained by resource subsidies provided by anthropogenic landscape change (Darlington et al., 2022; Fisher et al., 2021; Fuller et al., 2022), and it is acknowledged that white‐tailed deer are one of the primary prey sources for cougars in Alberta (Cooley et al., 2008; Knopff et al., 2010; Mallory et al., 2012; Smereka et al., 2020; Winkel et al., 2023). Thus, we cannot claim whether the lack of a strong relationship between cougars and white‐tailed deer activity is a true reflection of what is occurring on this landscape, if this represents some sort of threshold effect (e.g. due to white‐tailed deer activity being so widespread across all cameras), or if these signals went undetected due to limitations in cougar detections or the measure selected for prey activity. We also recognize that limitations in cougar detections could have resulted in undetected signals among other variables used in this study (see caveats). Further investigation is required to parse a part of the relationship between cougars, white‐tailed deer, and anthropogenic landscape development across the cougars' northeast range edge.

### 5.1 Caveats and limitations

We had a small sample size of independent cougar detections (*n* = 28) and site occurrences (*n* = 14), which constrained our model complexity (constricted to a maximum of two variables per model). Thus, our models were likely not representative of all the complex ecological interactions that exist within this study system. Besides moose, snowshoe hares, white‐tailed deer, and wolves, we did not include any other potential prey or competitor species in our analysis. There is intraguild predation and competition between cougars and mesopredators, such as coyote (*Canis latrans*) and lynx (*Lynx canadensis*; Engebretsen et al., 2021), and, due to the limitations of our sample size and detections, we did not include these species as the effects of these relationships can be difficult to parse apart. However, we provide here the basis for future studies to consider biotic interactions of multiple trophic levels (Srivathsa et al., 2023; Trainor et al., 2014), or explore alternative metrics for predator and prey activity across landscapes, to better understand and make predictions about cougar occurrence (Engebretsen et al., 2021). Here, we used relative activity indices which are comprised of the number of independent detections of a species divided by the number of active camera days, at a particular site. This, however, is not standardized for the differences in body sizes between prey items, or the differences in home range sizes that may inflate the number of individuals detected at a single site for smaller‐bodied prey.

We examined a range of spatial scales to capture differences in cougar resource selection between scales, however, we recognize that the results drawn from our spatial scales of choice (500, 2500 and 5000 m) may not apply to other spatial scales (Fisher et al., 2011). In ecological studies, biological responses are often not captured due to variables not measured at the effective scale of effect. Meta‐analysis of multi‐scale studies recommends increasing the number and range of scales being used in research, ideally with scales less than the size of the home range, to up to nine times the size of the average dispersal distance of the species being studied (Jackson & Fahrig, 2012, 2015). It is possible that we did not explore a wide enough range of spatial scales but were limited due to study design. Further, our inability to capture all relevant biological responses could have less to do with the scale of the buffers used and more to do with the extent of the sampling and the resolution at which prey activity was derived (i.e. a camera viewshed). The extent of our sampling may have captured more of a third‐order resource selection assessment, i.e., resource and habitat use within a few cougars' home ranges, rather than a range expansion assessment. We recognize that this is a preliminary study for cougars in this area, as there is little literature examining cougar occurrence in northeastern Alberta, and rigorous investigation through additional years of monitoring is still required.

## 6 CONCLUSIONS

After decades of persecution, cougars are expanding their range across eastern North America and have now been observed moving northward in the boreal forest (Anderson Jr. et al., 2009; Gantchoff et al., 2021; Knopff, Webb, & Boyce, 2014b; LaRue et al., 2012; Mallory et al., 2012; Morrison et al., 2015; Olson et al., 2021; Stoner et al., 2021; Winkel et al., 2023). We conclude that at this boreal range edge, cougars are primarily driven by native prey species, as observed in other studies (Knopff, Webb, & Boyce, 2014b; Maletzke et al., 2017), with a strong positive association with snowshoe hare activity. Although we did not detect a strong effect of white‐tailed deer activity and anthropogenic development features, parsing apart these relationships requires a more thorough investigation. As cougars recover and possibly move further into novel boreal landscapes, this could have unintended negative repercussions for endangered woodland caribou populations (Anderson Jr. et al., 2009). Understanding contemporary distributions and range shifts, especially for large carnivores, is integral to informing conservation management objectives for predator–prey dynamics and human concerns as large carnivore populations recover (Cimatti et al., 2021; Gantchoff et al., 2021; Moss, Alldredge, Logan, & Pauli, 2016a; Pratzer et al., 2023). We highlight the timely need to monitor and manage cougar populations and distributions at this northeast range edge as they continue to rebound, as we could be on the cusp of a major shift in the northern boreal community.

## AUTHOR CONTRIBUTIONS

**Millicent V. Gaston:** Formal analysis (supporting); writing – original draft (lead); writing – review and editing (equal). **Andrew F. Barnas:** Formal analysis (lead); writing – review and editing (equal). **Rebecca M. Smith:** Data curation (equal); writing – review and editing (equal). **Sean Murray:** Data curation (equal); writing – review and editing (supporting). **Jason T. Fisher:** Conceptualization (lead); supervision (lead); writing – review and editing (equal).

## CONFLICT OF INTEREST STATEMENT

The authors have no competing statement of interest to declare.

## Data Availability

All data are available at borealisdata.ca.

## APPENDIX A

**TABLE A1:** Beta coefficient table for the best‐supported model, Native Prey, predicting cougar (*Puma concolor*) occurrence across northeastern Alberta, Canada, at each spatial scale: 500, 2500 and 5000 m.

Scale		Estimate	Std. error	Z value	Pr(>|z|)
500, 2500, 5000 m^a^	Intercept	−1.1061	0.2870	−3.855	.000116
	Native Prey	0.5815	0.2679	2.170	.029980

^a^ Note values for “Native Prey” were identical at all three spatial scales, as this is a scale‐invariant predictor variable, therefore estimated effects are identical at all scales.
